# Diversification of OmpA and OmpF of *Yersinia ruckeri* is independent of the underlying species phylogeny and evidence of virulence-related selection

**DOI:** 10.1038/s41598-021-82925-7

**Published:** 2021-02-10

**Authors:** Michael J. Ormsby, Robert L. Davies

**Affiliations:** Institute of Infection, Immunity and Inflammation, College of Medical, Veterinary and Life Sciences, Sir Graeme Davies Building, University of Glasgow, 120 University Place, Glasgow, G12 8TA UK

**Keywords:** Genomic analysis, Genetics, Zoology, Microbiology, Bacteria, Bacterial genetics, Bacterial pathogenesis

## Abstract

*Yersinia ruckeri* is the causative agent of enteric redmouth disease (ERM) which causes economically significant losses in farmed salmonids, especially Atlantic salmon (*Salmo salar*) and rainbow trout (*Oncorhynchus mykiss*, Walbaum). However, very little is known about the genetic relationships of disease-causing isolates in these two host species or about factors responsible for disease. Phylogenetic analyses of 16 representative isolates based on the nucleotide sequences of 19 housekeeping genes suggests that pathogenic Atlantic salmon and rainbow trout isolates represent distinct host-specific lineages. However, the apparent phylogenies of certain isolates has been influenced by horizontal gene transfer and recombinational exchange. Splits decomposition analysis demonstrated a net-like phylogeny based on the housekeeping genes, characteristic of recombination. Comparative analysis of the distribution of individual housekeeping gene alleles across the isolates demonstrated evidence of genomic mosaicism and recombinational exchange involving certain Atlantic salmon and rainbow trout isolates. Comparative nucleotide sequence analysis of the key outer membrane protein genes *ompA* and *ompF* revealed that the corresponding gene trees were both non-congruent with respect to the housekeeping gene phylogenies providing evidence that horizontal gene transfer has influenced the evolution of both these surface protein-encoding genes. Analysis of inferred amino acid sequence variation in OmpA identified a single variant, OmpA.1, that was present in serotype O1 and O8 isolates representing typical pathogenic strains in rainbow trout and Atlantic salmon, respectively. In particular, the sequence of surface-exposed loop 3 differed by seven amino acids to that of other *Y. ruckeri* isolates. These findings suggest that positive selection has likely influenced the presence of OmpA.1 in these isolates and that loop 3 may play an important role in virulence. Amino acid sequence variation of OmpF was greater than that of OmpA and was similarly restricted mainly to the surface-exposed loops. Two OmpF variants, OmpF.1 and OmpF.2, were associated with pathogenic rainbow trout and Atlantic salmon isolates, respectively. These OmpF proteins had very similar amino acid sequences suggesting that positive evolutionary pressure has also favoured the selection of these variants in pathogenic strains infecting both species.

## Introduction

*Yersinia ruckeri* is the etiological agent of enteric redmouth disease (ERM) of cultured salmonids, causing significant economic losses to the fish-farming industry. First isolated in 1956 from diseased rainbow trout (*Oncorhynchus mykiss*, Walbaum)^1,2^, *Y. ruckeri* has since become widely disseminated and is present worldwide^3^, becoming increasingly responsible for infections in Atlantic salmon (*Salmo salar*). This is particularly the case in countries, such as Australia^4,5^, Chile^6,7^, Norway^8,9^, and Scotland^10^, where salmon production is of significant economic importance.

Extensive diversity has been demonstrated in isolates able to cause infection in Atlantic salmon and rainbow trout, with the identification of a novel emergent serotype in Atlantic salmon in recent years particularly striking^6,10–14^. Isolates recovered from rainbow trout are predominantly represented by serotype O1 and OMP-types 1b and 3a, while isolates recovered from Atlantic salmon are predominantly represented by serotypes O2, O5 and O8 and OMP-types 2a-c, 3a and 3b^10^. This diversity is supported by a more recent population study whereby isolates displayed degrees of host specificity, geographic endemism, and anthropogenic dissemination^12^. Subsequent work by Ormsby et al. examining outer membrane (OM) proteome composition revealed further variation between strains, providing insight into the potential roles of these diverse proteins in host–pathogen interactions^15^.

The importance of the OM in bacterial pathogenesis is well established, functioning as a physical barrier between the bacterial cell and its environment^16,17^. Outer membrane proteins (OMPs) comprise about 50% of the OM mass and function in OM biogenesis and integrity, transport, signal transduction, enzymatic activity and protection against antibiotics, detergents and toxins^17–20^. The OM is at the interface between pathogen and host, and OMPs play important roles in host–pathogen interactions including in adherence and colonisation, nutrient uptake, evasion of the host immune response and tissue damage^17^.

While the presence or absence of specific OMPs is one important aspect of variation between strains, further disparity within gene nucleotide sequences and the amino acid sequences of encoded proteins may contribute to host-specificity or lead to increased virulence. For example, some fish pathogenic bacteria, such as *Aeromonas hydrophila,* display antigenic diversity in OMPs while maintaining conservation of the OM proteome across serotypes^21^. Host-specific variation of surface-exposed proteins occurs in the type IV pili^22^, gonococcal porin^23^, transferrin-binding^24^ and lactoferrin-binding proteins^25^ of *Neisseria* sp.; the metallo-type IgA1 protease of *Streptococcus pneumoniae*^26^; and the avian-specific AC/I pili and lamb-specific K99 fimbriae of septicemic *Escherichia coli*^27,28^. In *Mannheimia haemolytica*, different variants of the major OM protein OmpA were associated with cattle and sheep suggesting a role in host-specificity^29^.

OmpA is one of the best studied and most well characterised OMPs. It is a multifunctional protein with roles in OM integrity^30^, as a non-selective pore^31^, as a bacteriophage receptor^32^, as an adhesin/invasin^33^, in immune evasion for macrophage survival^34^ and in biofilm formation^35^. OmpA forms an eight-stranded β-barrel in the OM of Gram-negative bacteria, with four surface-exposed loops and three periplasmic turns^36^. The surface-exposed loops of OMPs are involved in many host and environmental interactions^37–40^. The role of OmpA in host-specificity has been suggested previously; variation in the protein sequence of OmpA is thought to influence the virulence and host-specificity of extraintestinal pathogenic *E. coli* (ExPEC) subpathotypes^41^; while significant host-specific variation has been observed in the OmpA sequence of isolates of M. *haemolytica*^29^*.* Due to the presence of OmpA in such high abundance within the OM^18^, its role in host-specifity and the numerous functions elicited are not surprising.

Together with OmpA, the major porin OmpF forms the basis of the *Y. ruckeri* OMP-typing scheme^42^. Porins are, by definition, non-specific transmembrane β-barrel structures forming channels across the bacterial membrane^16^. They allow the diffusion of small (< 600 Da), hydrophilic molecules through aqueous pores and in general show no particular substrate specificity, despite some selectivity for cations or anions^16,18^. Transcription of the major porin *ompF* is regulated by several environmental factors including osmolarity, temperature, pH, nutrient availability, aeration and various toxins^43–46^. The large size of OmpF (pore diameter ranging from 6 Å for the highly selective porins to 15 Å for the general porins^19^) is attributed to improving the efficiency of nutrient uptake in a nutritionally poor environment^45,47^. Variation in the loop regions of OmpF have been described in many species, including *Yersinia *sp.^48^, *E. coli*^49^ and *Salmonella Typhi*^50^. The loops of OmpF are known to have putative antigenic epitopes^51,52^ and indeed, a recombinant OmpF subunit vaccine has been shown to provide protective immunity against *Y. ruckeri* infection in catfish^53^.

The aim of this study was to explore the phylogenetic relationship of 16 representative isolates of *Y. ruckeri* based on nucleotide sequence variation within 19 housekeeping genes and, subsequently, to assess variation in the nucleotide and amino acid sequences of the major OMPs OmpA and OmpF. In this way, we aimed to establish whether these proteins displayed specificity towards rainbow trout and Atlantic salmon as this could indicate a role in virulence and offer a potential route for preventing infection.

## Materials and methods

### Bacterial strains and culture conditions

Sixteen isolates of *Y. ruckeri* recovered from Atlantic salmon (7 isolates), rainbow trout (8 isolates) and European eel (*Anguilla anguilla*; 1 isolate) and from diverse geographic locations (U.K.; Germany; Italy; Denmark; Finland) were included in this study. The isolates represented a range of biotypes, serotypes, OMP-types and also allowed isolates of similar phenotypes recovered from different time periods to be compared. These isolates have been extensively characterised, including those collected pre-1990^14,54^, together with contempary isolates^10^. The properties of the isolates used are presented in Table 1. Bacteria were stored at − 80 °C in 50% glycerol (*v/v*) in tryptone soya broth (TSB; Oxoid) and were routinely subcultured on tryptone soya agar (TSA; Oxoid) at 22 °C for 48 h. Liquid cultures were prepared by inoculating three or four colonies into 10-ml volumes of TSB and incubating overnight at 22 °C with shaking at 120 rpm.

**Table 1:** Properties of *Yersinia ruckeri* isolates included in the sequencing study.

Lab designation	Previous designation	Date isolated	Origin		Phenotypic characteristics			Molecular characteristics			
			Geographic	Host species	Biotype	Serotype	OMP type	OmpA	*ompA*	OmpF	*ompF*
RD6	–	Pre 1990	Scotland	Rainbow trout	2	O1	1b	OmpA1	*ompA1.1*	OmpF1	*ompF1.1*
RD10	–	1985	Scotland	Rainbow trout	2	O1	1a	OmpA1	*ompA1.1*	OmpF1	*ompF1.1*
RD28	BA2	Pre 1990	U.K	Rainbow trout	1	O5	2a	OmpA2	*ompA2.1*	OmpF6	*ompF3.4*
RD64	F53.1/82	1982	West Germany	Rainbow trout	1	O2	2a	OmpA2	*ompA2.1*	OmpF5	*ompF3.3*
RD84	5710/83	1983	Italy	Rainbow trout	1	O1	3b	OmpA1	*ompA1.1*	OmpF1	*ompF1.1*
RD124	851,014	Pre 1990	Denmark	Rainbow trout	1	O1	3a	OmpA1	*ompA1.1*	OmpF1	*ompF1.1*
RD150	38/85	1985	Denmark	European Eel	1	O7	1a	OmpA3	*ompA3.1*	OmpF7	*ompF3.5*
RD162	–	Pre 1990	Finland	Atlantic salmon	1	O6	2c	OmpA2	*ompA2.2*	OmpF3	*ompF3.1*
RD290	–	Pre 1990	Scotland	Atlantic salmon	1	O5	2c	OmpA2	*ompA2.3*	OmpF2	*ompF2.2*
RD354	TW60/05	20/05/2005	Scotland	Atlantic salmon	1	O2	2a	OmpA2	*ompA2.1*	OmpF4	*ompF3.2*
RD366	TW90/05	20/07/2005	Scotland	Atlantic salmon	1	O5	2c	OmpA2	*ompA2.3*	OmpF2	*ompF2.2*
RD382	FVG 269/06	06/05/2006	U.K	Atlantic salmon	1	O1	3a	OmpA1	*ompA1.2*	OmpF2	*ompF2.1*
RD420	TW110/08	08/07/2008	Outer Hebrides	Atlantic salmon	1	O8	3a	OmpA1	*ompA1.2*	OmpF2	*ompF2.1*
RD520	FVG 205 (T1-1)	26/04/2011	Scotland	Rainbow trout	2	O1	1a	OmpA1	*ompA1.1*	OmpF1	*ompF1.1*
RD524	TW87/11	21/07/2011	E Scotland	Rainbow trout	2	O8	1a	OmpA1	*ompA1.1*	OmpF1	*ompF1.1*
RD532	TW119/11	28/09/2011	Scotland	Atlantic salmon	1	O8	3a	OmpA1	*ompA1.2*	OmpF2	*ompF2.1*

### Extraction of genomic DNA

Genomic DNA was extracted from overnight liquid cultures using the PurElute Bacterial Genomic DNA Kit (Edgebio; 85171) according to the manufacturer’s instructions. The DNA was resuspended in 100 µl nuclease-free water and stored at − 20 °C.

### Whole genome sequencing

Whole genome sequencing was conducted using the Illumina Mi-seq system at Glasgow Polyomics, employing 300-bp paired-end sequencing. Reads were assembled using CLC Genomics workbench version 7.5.2 (Qiagen). Reads were trimmed for contaminating sequence adapters and low quality bases removed with a threshold of a shred score of 25. Reads shorter than 150 bp after quality trimming were discarded. Assembled scaffolds were annotated with Rapid Annotation using the Subsystem Technology (RAST) resource^55,56^. Raw reads have been deposited at the European Nucleotide Archive (ENA) under accession number PRJEB40607. Genome coverage information is included as Supplementary Table S1.

### Phylogeny of *Y. ruckeri*

**Figure 1:**
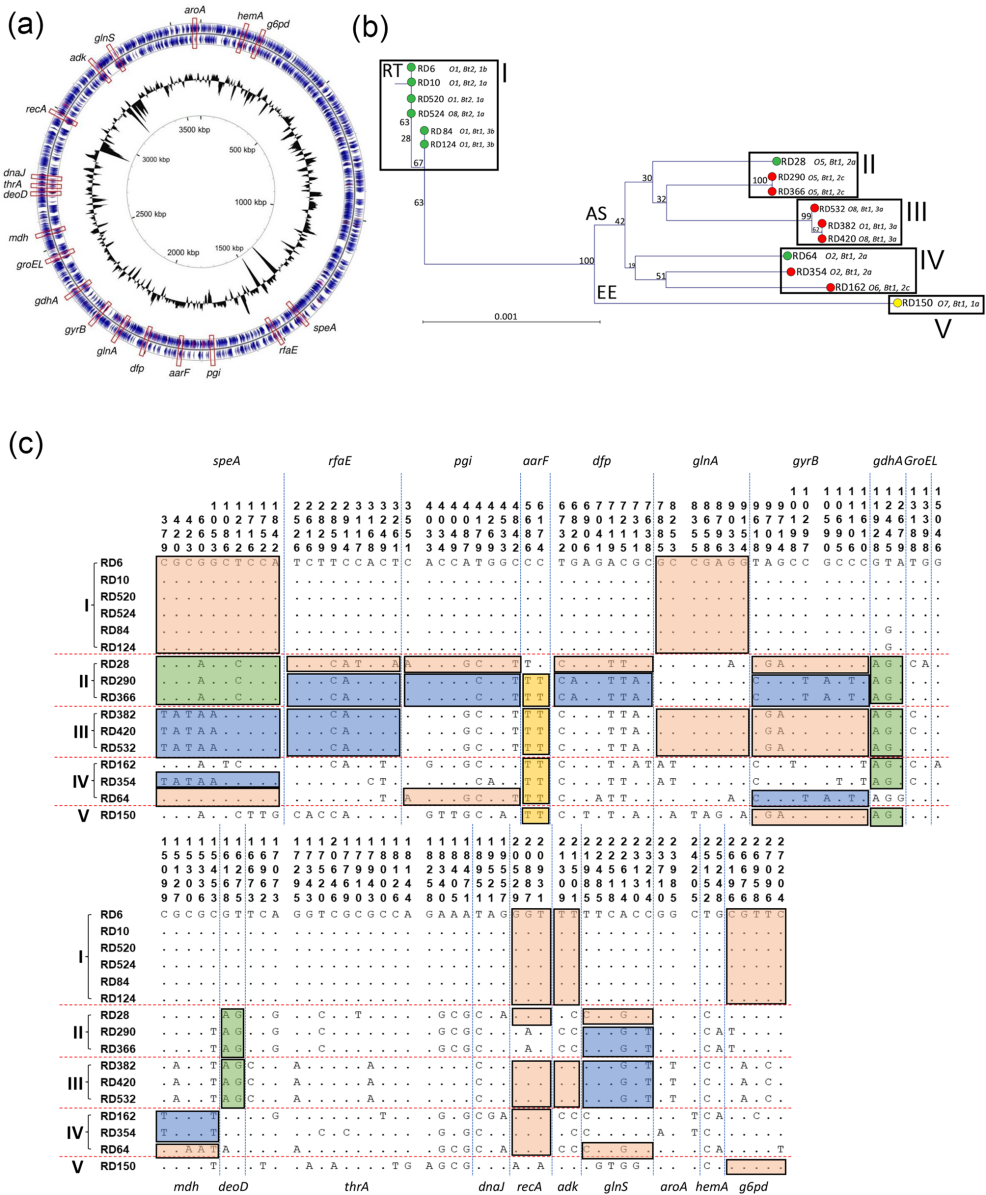
Location of housekeeping genes used for MLST on the closed chromosome of *Y. ruckeri* strain YRB and phylogenetic relatedness amongst representative isolates. (**a**) The positions of the nineteen housekeeping enzymes used to construct phylogeny of *Y. ruckeri* within the closed genome of strain YRB (GenBank: CP009539.1) are shown. The circular genome map was generated using CGView Server software (V 1.0)^60^ and annotated using Microsoft PowerPoint. (**b**) A Neighbour joining phylogenetic tree shows the relationship of 16 isolates (Table 1) based on the concatenated sequence of the 19 housekeeping genes (Table 2). This concatenated sequence was 27,363 nucleotides in length and encoded 9121 amino acids. Isolates were recovered from Atlantic salmon (AS; red); rainbow trout (RT; green); and European eel (EE; yellow). Parameters were assessed with a bootstrap method (1000 tests). The tree was constructed using the Jukes–Cantor correction for synonymous changes. Distribution of polymorphic nucleotide sites among concatenated housekeeping gene sequences in reference isolates (**c**). The numbers above the sequences represent the positions of polymorphic nucleotide sites from the 5′ end of the gene. The dots represent sites where the nucleotides match those of the first sequence (RD6). The boxes highlight regions of sequence identity that represent proposed recombinant segments. Genes within the concatenated sequence are separated by dashed lines. Roman numerals correlate with positions on the phylogenetic tree in (**b**).

The nucleotide sequences of 19 housekeeping genes, distibuted throughout the chromosome to remove bias or selection of a single area that may have undergone extensive recombination, were obtained from reference strain ATCC 29473. The genes selected were included in previous MLST studies of *Y. ruckeri*^11^ and other members of the *Yersinia* genus^57,58^. The properties of these genes are summarised in Table 2 and their positions within the genome are indicated in Fig. 1a. The same 19 gene sequences were extracted from the genomes of the 16 representative isolates of *Y. ruckeri* using the CLC genomics ‘internal Blast’ tool and concatenated using the CLC Genomics ‘join sequences’ tool. A concatenated sequence of 27,363 nucleotides in length was generated for each isolate. Using the BLAST tool in CLC, genes were considered to match when sequence coverage was > 98%.

**Table 2:** Housekeeping genes included in phylogenetic study of *Yersinia ruckeri.*

Gene	Name	Position in reference genome (ATCC 29473)		Direction	Length (nt)
		Start	Stop		
*aarF*	Ubiquinone biosynthesis	1,853,520	1,855,154	Complement	1634
*adk*	Adenylate kinase	3,172,292	3,172,936	Forward	644
*aroA*	3-Phosphosphikimate 1-carboxyvinyltransferase	3,571,559	3,572,845	Forward	1286
*deoD*	Purine nucleoside phosphorylase	2,713,635	2,714,360	Forward	725
*dfp*	Phosphpantothenate-cysteine ligase	2,094,362	2,095,576	Forward	1214
*dnaJ*	Chaperone protein	2,747,109	2,748,242	Forward	1133
*g6pd*	Glucose-6-phosphate 1-dehydrogenase	212,504	213,979	Forward	1475
*gdhA*	Quinoprotein glucose dehyrdogenase A	2,313,006	2,314,349	Complement	1343
*glnA*	Glutamine synthetase	2,025,330	2,026,739	Forward	1409
*dlnS*	Glutamine-tRNA ligase	3,298,889	3,300,556	Forward	1667
*groEL*	60 kDa chaperonin	2,476,907	2,478,550	Forward	1643
*gyrB*	DNA gyrase subunit B	2,204,186	2,206,600	Forward	2414
*hemA*	Glutamyl-tRNA reductase	160,404	161,666	Forward	1262
*mdh*	Malate dehydrogenase	2,549,973	2,550,908	Forward	935
*pgi*	Glucose-6-phosphate 1-isomerase	1,736,929	1,738,575	Complement	1646
*recA*	Recombinase A	2,990,962	2,992,032	Forward	1070
*rfaE*	Bifunctional heptose 7-phosphate kinase/heptose 1-phosphate adenyltransferase	1,512,212	1,513,642	Complement	1430
*speA*	Biosynthetic arginine decarboxylase	1,410,853	1,412,832	Complement	1979
*thrA*	Bifunctional aspartokinase/homoserine dehydrogenase 1	2,732,281	2,734,740	Forward	2459
				Total:	27,368

Genes were analysed using CLC Genomics in conjunction with alignment programs written by T. S. Whittam (Michigan State University). Sequences were firstly formatted using Microsoft Word (Microsoft, Office 2010) to remove line breaks and correct spacing issues. Finalised nucleotide sequences were converted into amino acid sequences using AASEQ (T.S.W.) and the amino acid sequences aligned using ClustalX (Conway Institute, UCD). The nucleotide sequences were subsequently aligned based on the amino acid alignments using REALIGX (T.S.W.). A phylogenetic Neighbor-Joining relatedness tree with Jukes-Cantor correction for synonymous changes was constructed from the 19 concatenated sequences using MEGA (version X); boot-strap analysis was performed with 1000 replicates. The distribution of polymorphic nucleotide sites within alleles was represented graphically using the programmes PSFIND and HAPPLOT (T.S.W.) in conjunction with Graphics Suite^2^ (Micrografx) Designer. Split decomposition trees of the housekeeping genes, *ompA* and *ompF* were constructed with SplitsTree 4.0 (http://www.splitstree.org) to test for recombination^59^. Splits decomposition analysis is used to identify ‘net-like phylogeny’ which is characteristic of recombination.

### Nucleotide and amino acid sequence analysis of OmpA and OmpF of *Y. ruckeri*

Using the ‘internal Blast database’ created as described above and sequences from ATCC 29473 as a template, gene sequences of *ompA* and *ompF* were identified, and extracted from the 16 isolates for phylogenetic analyses using CLC Genomics Workbench. Phylogeny was determined using CLC Genomics and alignment programmes, as previously described. Neighbour-Joining phylogenetic trees (Jukes-Cantor model) were generated to determine sequence variation and boot-strap analysis (1000 replications) was conducted.

### Structural analysis of OmpA and OmpF

Secondary structure predictions were performed using Pred TMBB^61^. Structures were based on sequences from *Y. ruckeri* reference isolate ATCC 29473. Molecular weights of OmpA and OmpF were calculated using the ExPASY Compute pI/Mw tool^62^.

## Results and discussion

Serotypic diversity and host-specificity have been demonstrated previously in isolates of *Y. ruckeri*^10,12,15^. While isolates of serotype O1 are the major cause of disease in rainbow trout worldwide, the emergence of novel serotypes coupled with reduced vaccine efficacy have resulted in an increased variety of isolates infecting other species, particularly Atlantic salmon. This study aimed to elucidate the underlying genetic relatedness of a selection of *Y. ruckeri* isolates from Atlantic salmon and rainbow trout based on nucleotide sequence variation in 19 housekeeping genes. Against the phylogenetic background so derived, we then wished to explore the molecular evolution and diversity of the OmpA and OmpF proteins in the same isolates with a view to further understanding their potential roles in host-adaptation and virulence. OmpA and OmpF form the basis of the *Y. ruckeri* OMP-typing scheme^42^, were two of the most abundant OMPs identified in our previous proteomic analysis^15^, and are known to play various roles, via variation in their surface-exposed loops, in pathogenesis in other pathogens^51,63–73^.

### Evidence for recombination and distinct host-specific lineages in *Y. ruckeri*

The concatenated sequence representing the 19 housekeeping genes of *Y. ruckeri* (Table 2; Fig. 1a) was 27,363 nucleotides in length and encoded 9121 amino acids. A phylogenetic tree constructed from the 16 concatenated sequences was represented by five distinct clusters with isolates recovered from Atlantic salmon, rainbow trout and European eel mostly residing on separate branches (Fig. 1b). Splits decomposition analysis of the 16 unique housekeeping gene sequences produced a network suggestive of a degree of recombination (Supplementary Fig. S1a). Although we studied fewer isolates, the clustering observed here (Fig. 1b) is nevertheless similar to that described by Gulla et al. in which isolates recovered from different host species clustered together into closely related clonal complexes. Isolates recovered from rainbow trout and Atlantic salmon were, with two exceptions, phylogenetically distinct and were represented in the RT (I) and AS (II, III and IV) clusters, respectively. The two exceptions were the rainbow trout isolates RD28 and RD64 which were closely related to the Atlantic salmon isolates, demonstrating evidence of mosaicism and recombinational exchange. The serotype O7 European eel isolate, RD150, represented a distinct lineage on the tree in cluster V (Fig. 1b, EE cluster). The branching of the European eel isolate (as a reference serotype O7 strain) suggests an earlier host-associated evolutionary split within *Y. ruckeri*, although the relativelty low sample number should be considered with further analysis needed to support this observation.

Within cluster I, all isolates were recovered from rainbow trout (Fig. 1b, RT). The biotype 1, serotype O1 isolates RD84 and RD124 are typical ‘Hagerman’ isolates and clustered together on the tree. Similarly, the non-motile biotype 2, serotype O1 variant represented by strains RD6, RD10 and RD520 also clustered together and are very closely related to isolates RD84 and RD124. The novel serotype O8 isolate RD524 is identical to the serotype O1, biotype 2 isolates RD6, RD10 and RD520, suggesting that this isolate has evolved from these and not from the ‘Hagerman’ strain (isolates RD84 and RD124). Closer inspection of the 19 housekeeping gene sequences revealed complete identity in 18 out of 19 genes between these six isolates (RD6, 10, 520, 524, 84 and 124), with only one polymorphic nucleotide site present in *gdhA* of isolates RD84 and RD124 (Fig. 1c and Supplementary Fig. S2). These isolates represent the most virulent clone(s) of *Y. ruckeri* circulating within the rainbow trout population and their uniformity is likely due to rapid epidemic clonal expansion as has been suggested for French isolates of *Y. ruckeri* and observed in the fish pathogen *Flavobacterium psychrophilum*^74–76^*.*

The Atlantic salmon isolates are represented by three clusters (II, III and IV) on the phylogenetic tree (Fig. 1b, AS). In cluster II, the serotype O5 Atlantic salmon isolates RD290 and RD366 are identical, but notably distinct from the serotype O5 rainbow trout isolate RD28. However, inspection of the concatenated housekeeping gene sequence revealed evidence of horizontal gene transfer within this cluster. Whereas the three isolates (RD28, RD290 and RD366) were identical, or nearly identical, at 13 loci (*speA, aarF, glnA, gdhA, mdh, deoD, thrA, dnaJ, recA, adk, aroA, hemA* and *g6pd*), there was evidence that isolate RD28 has diverged more widely from isolates RD290 and RD366 potentially by exchange of alleles at 6 loci (*rfaE*, *pgi*, *dfp*, *gyrB, groEL* and *glnS*). In particular, the *gyrB* allele of RD28 was very different to that of RD290 and RD366 but identical to that of Atlantic salmon isolates RD382, RD420 and RD532 (cluster III). Similarly, the *pgi* and *glnS* alleles of RD28 also differ from those of isolates RD290 and RD366, but are identical to those of rainbow trout serotype O2 isolate RD64 (cluster IV). The complete identity of alleles at these three loci among these otherwise divergent isolates clearly demonstrate common origins.

In Cluster III, the Atlantic salmon serotype O1 isolate RD382, and serotype O8 isolates RD420 and RD 532, are identical at all 19 loci, with the exception of a single nucleotide polymorphism in *groEL*. However, these isolates share alleles of 100% identity with either two (*rfaE* and *glnS*; RD290 and RD366) or three (*gdhA* and *deoD*; RD28, RD290 and RD366) isolates in cluster II, whereas other alleles are divergent in isolates from these two clusters which accounts for the deep branching of cluster III (e.g. speA, gyrB, thrA and g6pd). Again, these data suggest exchange of alleles by horizonatl gene transfer between strains in clusters II and III.

Cluster IV comprises the Atlantic salmon serotype O2 and O6 isolates RD354 and RD162, together with the rainbow trout serotype O2 isolate RD64. These three isolates represent distinct branches and RD354 and RD162 are more closely related to each other than to the other Atlantic salmon isolates (Fig. 1b). The isolates share complete identity at the *aarF* and *recA* loci (both with each other and with other strains) but they are divergent at most of the other loci (which accounts for the deep branching of the isolates within this cluster). However, isolate RD64 shares complete, or near complete, identity with the other rainbow trout isolates of cluster 1 at the *speA* and *g6pd* loci and with isolate RD28 at *pgi* and *glnS.* Taken together, these results suggest that isolate RD64 possibly has an Atlantic salmon ancestor, but has acquired certain rainbow trout alleles after switching hosts.

Cluster V represents a single lineage (EE) comprising the serotype O7 European eel isolate RD150 (Fig. 1b). Although RD150 is divergent to all other isolates at a number of loci (*speA, rfaE, pgi, dfp, glnA, thrA and glnS*), it also shares complete identity with various isolates at other loci. Thus, RD150 is identical at the *aarF* locus with isolates of clusters II, III and IV, at *gyrB* with isolates of clusters II and III, at *gdhA* with isolates of clusters II, III and IV, at *deoD* with isolates of clusters I and IV, at *dnaJ* with isolates of cluster I, at *adk* with isolates of clusters I and III, and at *g6pd* with isolates of clusters I, II and IV. Thus, isolate RD150 has a more complex phylogeny with genes having ancestral origins in both rainbow trout and Atlantic salmon isolates.

The very close identities of the serotype O1 RD382 and serotype O8 RD420 Atlantic salmon isolates suggest that they have a common evolutionary ancestor (Fig. 1b). Similarly, the very close identities of the serotype O1 biotype 2 RD6/RD10/RD520 and serotype O8 biotype 2 RD524 rainbow trout isolates also suggests a common evolutionary origin. In both cases, it is reasonable to speculate that the recently-identified serotype O8 isolates have emerged from serotype O1 isolates as a result of the acquisition of a gene, or genes, involved in the biosynthesis of the new O8 O-antigen type by horizontal gene transfer (HGT)^10^. We have previously hypothesised that the O8 serotype most likely arose in Atlantic salmon because it was first identified, and is far more abundant, in Atlantic salmon isolates. The serotype O8 phenotype first appeared in Atlantic salmon isolates in 2002 before becoming the dominant serotype by 2006; in contrast, this serotype was not identified in rainbow trout isolates until 2010^10^. The identification of the O8 serotype in genetically divergent Atlantic salmon and rainbow trout isolates provides strong support for a second subsequent and more recent evolutionary event; that is, genetic exchange of the O8 O-antigen biosynthesis gene cluster from (most likely) Atlantic salmon to rainbow trout isolates, as previously suggested^10^. However, it is also plausible that this serotype may have emerged independently, in a separate gene acquisition event, in rainbow trout. Previously, Ormsby et al. speculated that the widespread use of serotype O1-based vaccines in Scotland may account for the emergence of the O8 serotype in Scottish Atlantic salmon^10^.

Using multilocus variable-number tandem-repeat analysis (MLVA), Gulla et al. demonstrated that serotype O8 isolates recovered from Atlantic salmon were found within a clonal complex that was predominantly associated with isolates of serotype O1. These observations were explained by recombinational events involving the lipopolysaccharide (LPS) biosynthesis cascade suggestive of vaccine-related evolutionary pressures similar to those associated with the independent emergence of the various *Y. ruckeri* biotype 2 lineages^77–79^. Horizontal transfer of the LPS biosynthesis gene cluster (complete or partial), including genes involved in O-antigen biosynthesis, is not uncommon in Gram-negative bacteria. Thus, O-antigen biosynthesis gene clusters have been horizontally acquired by *A. hydrophila* strains responsible for septicemia in catfish, contributing to LPS diversity^80^; discrete regions of sequence diversity have been observed in the O-antigen locus of *Bordetellae*, again suggestive of HGT between strains^81^; and, switching of the O-antigen gene cluster between strains of *E. coli* that colonize cattle has also been recently described^82^. Therefore, it is reasonable to speculate that similar processes have been involved in the emergence of the O8 O-antigen in divergent Atlantic salmon and rainbow trout *Y. ruckeri* strains.

### Variation in the amino acid sequence of OmpA may relate to *Y. ruckeri* virulence

**Figure 2:**
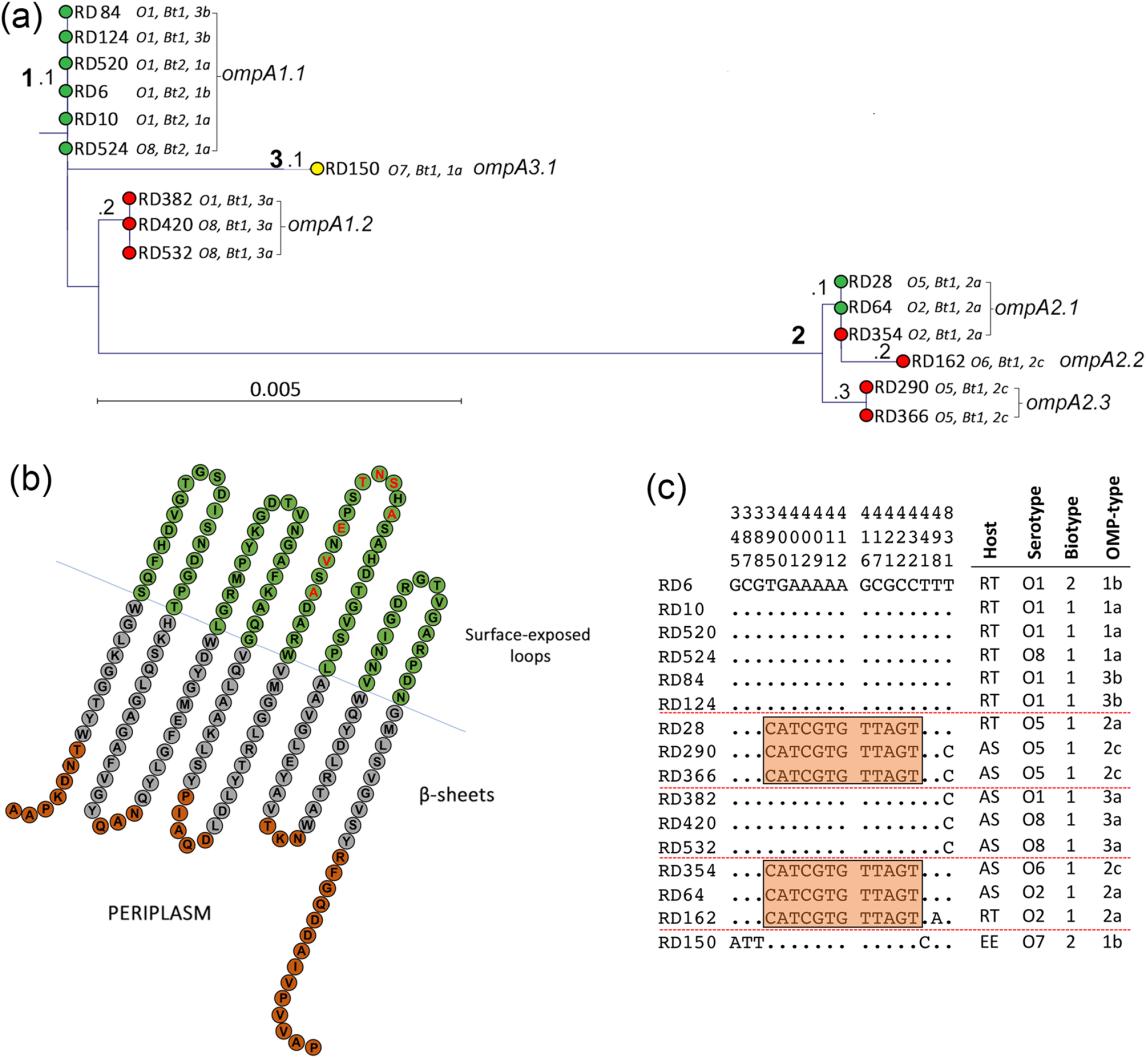
Variation with the protein sequence of OmpA is confined to a hypervariable region of loop 3. (**a**) Phylogenetic relatedness of *ompA* amongst 16 isolates of *Y.* ruckeri. Isolates were recovered from rainbow trout (green), Atlantic salmon (red) and European eel (yellow). The tree was constructed using the Jukes–Cantor correction for synonymous changes. Distinct variants are indicated by i–iii and reflect Supplementary Fig. S1. (**b**) The proposed secondary structure the OmpA protein of *Y. ruckeri*. The sequence is based on OmpA of strain ATCC29473. The periplasmic turns are shown in red, the β-sheets in grey and the surface exposed loops in green. Variable amino acids are shown in bold red and correspond with the sequence alignment in Supplementary Fig. S1. Domains were predicted using Pred TMBB based on Hidden Markov Models (HMM). Sequences at both N- and C-terminals are truncated. Distribution of polymorphic nucleotide sites among *ompA* alleles in reference isolates (**c**). The numbers above the sequences represent the positions of polymorphic nucleotide sites from the 5′ end of the gene. The dots represent sites where the nucleotides match those the first sequence (RD6). The boxes highlight regions of sequence identity that represent proposed recombinant segments.

Nucleotide sequence variation of the *ompA* gene from the sixteen isolates was minimal. Only six alleles were identified (*ompA1.1* and *ompA1.2*, *ompA2.1* to *ompA2.3* and *ompA3.1*; Fig. 2a) and there were only 18 polymorphic nucleotide sites in a gene 1065 nucleotides in length (1.69%) and seven variable inferred amino acid sites out of 355 (1.97%; Fig. 2a and Supplementary Fig. S1). The rainbow trout serotype O1 and O8 isolates RD6, RD10, RD84, RD124, RD520 and RD524 had identical *ompA* sequences (allele *ompA1.1*; Fig. 2a, cluster [1]), which were very similar to those of the Atlantic salmon serotype O1 and O8 isolates RD382, RD420 and RD532 (*allele ompA1.2*; Fig. 2a, cluster [1]). The *ompA* sequence of serotype O5 rainbow trout isolate RD28 was identical to those of serotype O2 isolates RD64 and RD354 recovered from rainbow trout and Atlantic salmon, respectively (*ompA2.1*; Fig. 2a, cluster [2]). These isolates had *ompA* sequences very closely related to those of the Atlantic salmon serotype O5 strains RD290 and RD366 (*ompA2.3*; Fig. 2a, cluster [2]). The *ompA* sequence of serotype O6 strain RD162 was similar to the *ompA* sequence of the serotype O2 and O5 isolates (*ompA2.2*; Fig. 2a, cluster [2]), while the *ompA* sequence of serotype O7 isolate RD150 was most similar to the serotype O1 and O8 isolates, although it represented a distinct lineage on the tree (*ompA3.1*; Fig. 2a, cluster [2]).

Comparison of the the phylogenetic relatedness trees of the housekeeping genes (Fig. 1b) and *ompA* (Fig. 2a) reveals that the two trees are non-congruent. Thus, the *ompA* sequences of the Atlantic salmon isolates RD382, RD420 and RD532 are very closely related to the *ompA* sequences of the rainbow trout isolates RD84, RD124, RD520, RD6, RD10 and RD524 (Fig. 2a); in contrast, the Atlantic salmon and rainbow trout isolates are themselves relatively highly diverged based on their housekeeping enzyme genes (Fig. 1b). The close similarity of *ompA* in these Atlantic salmon and rainbow trout isolates strongly suggests that either part of *ompA*, or the entire gene, has been horizontally transferred. The 100% identity of *ompA* in isolates RD28, RD64 and RD354 (Fig. 2a) is also at odds with the diverged relationships of these three strains based on their housekeeping gene sequences (Fig. 1b) and is also suggestive of HGT events influencing the distribution of *ompA*. Based on the phylogeny of their housekeeping genes (Fig. 1b), the isolates are generally clustered according to their serotypes and the host species of origin. In contrast, the *ompA* phylogeny reveals that the isolates are clustered together based, to a large extent, on their OMP-types. Thus, clusters of identical *ompA* sequences are associated with OMP-types 1a, 1b and 3b, 3a, 2a, and 2c, respectively. These findings are not surprising because the OMP-typing scheme is itself partially based on molecular mass variation of OmpA and, to some extent, provide validation of the usefulness of the OMP-typing approach.

Upon inspection of the OmpA amino acid sequence variation (Supplementary Fig. S3) and identification of the loop and β-barrel transmembrane domains in the protein, three distinct variants were identified (Fig. 2b). These were designated OmpA.1 to OmpA.3 and reflect clusters 1, 2 and 3 of the *ompA* phylogenetic relatedness tree (Fig. 2a). *Yersinia ruckeri* strains may be differentiated based on molecular mass variation of the major OMPs OmpA, OmpC and OmpF^35^ and the calculated molecular mass of OmpA similarly varied in each of the clusters. Thus, the molecular mass of OmpA was 37,755, 37,771 and 37,793 Da in isolates of clusters 1–3, respectively. Noteably, the amino acid variation occurred in loop 3 of the protein; thus, loop 3 differed at six amino acid sites in OmpA.1 and OmpA.2 (Fig. 2b and Supplementary Fig. S1). This involved amino acid substitutions at sites V132A, E134I, T137A, N138D, S139I and A141R. The extensive diversity in loop 3 (19.7% nucleotide variation and 27.3% amino acid variation) was in stark contrast to the absence of diversification in the membrane spanning regions of the protein (0.9% nucleotide variation and 0% amino acid variation). Previous studies have described OmpA as an important adhesin^63,66^, including in fish pathogenic bacteria^67^. Loops 1, 2 and 3 have been demonstrated to play important roles in invasion and adherence of *E. coli*^68^. Extensive variation has been identified in OmpA of extraintestinal pathogenic *E. coli* (ExPEC), in which 25 different polymorphism patterns were identified, with particular variation identified in loop 3^41^. However, our analysis of OmpA amino acid sequence variation suggests that the OmpA protein of *Y. ruckeri* is unlikely to play a role in host specificity. The Atlantic salmon and rainbow trout isolates present in cluster 1 were recovered from diseased fish and represent virulent clones of *Y. ruckeri*. However, the 100% identity of OmpA and, in particular, the lack of variation in the loop regions in these isolates, likely precludes a role in host-specificity (such as in binding to host-specific receptor molecules) unless post-translation protein modifications occur. Nevertheless, the presence of a conserved OmpA type (OmpA.1) in Atlantic salmon and rainbow trout isolates of serotypes O8 and O1, respectively, that are responsible for the majority of disease in these host species^10,83,84^ does suggest a potential role for this protein in virulence. Finally, the identical OmpA sequences shared by serotype O1 and O8 isolates recovered from Atlantic salmon and rainbow trout, respectively, provides additional evidence that the novel O8 phenotype has arisen independently in these species from different serotype O1 strains.

### Variation in the sequence of OmpF may promote immune evasion and adhesion

**Figure 3:**
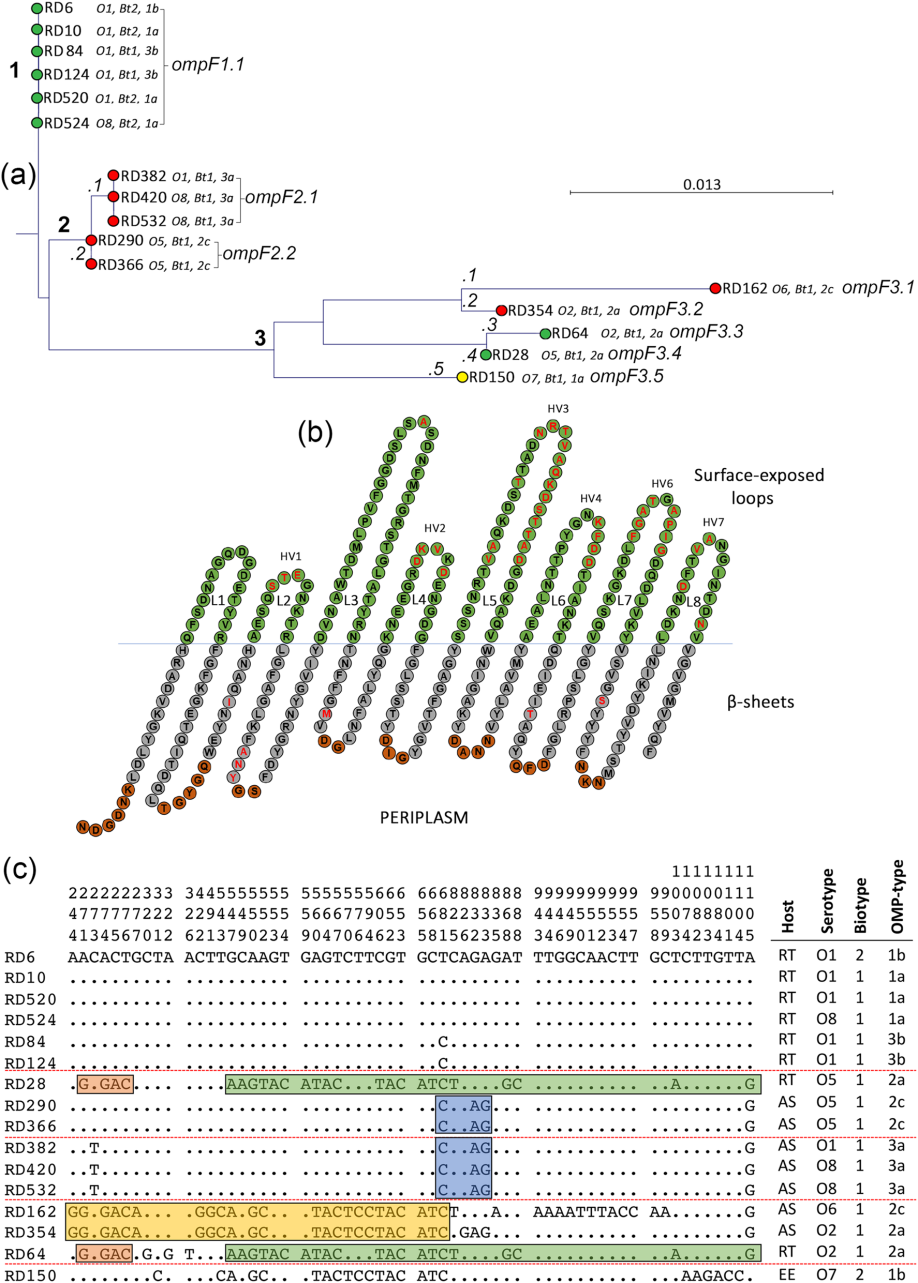
Variation with the protein sequence of OmpF is confined to hypervariable regions of the loops. (**a**) Phylogenetic relatedness of *ompF* amongst 16 isolates of *Y.* ruckeri. Isolates were recovered from rainbow trout (green), Atlantic salmon (red) and European eel (yellow). The tree was constructed using the Jukes–Cantor correction for synonymous changes. Distinct variants are indicated by i–vii and reflect Supplementary Fig. S2. (**b**) The proposed secondary structure the OmpF protein of *Y. ruckeri*. The sequence is based on OmpF of strain ATCC29473. The periplasmic turns are shown in red, the β-sheets in grey and the surface exposed loops in green. Variable amino acids are shown in bold red and correspond with the sequence alignment in Supplementary Fig. S2. Domains were predicted using Pred TMBB based on Hidden Markov Models (HMM). Sequences at both N- and C-terminals are truncated. Distribution of polymorphic nucleotide sites among *ompF* alleles in reference isolates (**c**). The numbers above the sequences represent the positions of polymorphic nucleotide sites from the 5′ end of the gene. The dots represent sites where the nucleotides match those the first sequence (RD6). The boxes highlight regions of sequence identity that represent proposed recombinant segments.

Substantially greater nucleotide and amino acid variation was observed in the porin OmpF (Fig. 3; Supplementary Fig. S2) than in OmpA (Fig. 2; Supplementary Fig. S1). Eight alleles were identified which were designated *ompF1.1*, *ompF2.1* and *ompF2.2* and *ompF3.1* to *ompF3.5* (Fig. 3a). The *ompF* gene was 1140 nucleotides in length and the predicted protein comprised 379 amino acids; there were 103 (9.0%) polymorphic nucleotide sites and 47 (12.4%) variable inferred amino acid positions among the *ompF* alleles. The phylogenetic relationships of the *ompF* sequences are shown in Fig. 3a. and three major clusters (1–3) were distinguished. Cluster 1 comprises the six serotype O1 and O8 rainbow trouts isolates (RD6, RD10, RD84, RD124, RD520 and RD524 [*ompF1.1*]; Fig. 3a). Noteably, the biotype 2 isolates RD6, RD10, RD520 and RD524 could be distinguished from the biotype 1 isolates RD84 and RD124 in having a 13-amino acid insertion within loop 5 (due to the insertion of 39 additional nucleotides between positions 684 and 720 in the *ompA* gene); this resulted in an increase in molecular mass of OmpF from 40,198 to 41,586 Da in these isolates (Supplementary Fig. S2). Indeed, this variation in OmpF is observable in SDS–polyacrylamide gels and is utilised in the OMP-typing scheme^42^. These differences were not evident in the phylogenetic tree which did not take insertions/deletions into account. Isolates within cluster 1 were of OMP-types 1a, 1b and 3b. Cluster 2 comprises the five Atlantic salmon isolates of serotypes O1, O5 and O8 (RD382, RD420, RD532, RD290 and RD366). The serotype O1 Atlantic salmon isolate RD382 clustered with the serotype O8 isolates RD420 and RD532 (*ompF2.*1 and OMP-type 3a) and were closely related to the serotype O5 Atlantic salmon isolates RD290 and RD366 (*ompF2.2* and OMP-type 2c). The encoded OmpF protein (OmpF.2) in these Atlantic salmon isolates had identical amino acid sequences and a molecular mass of 40,024 Da. Together, these Atlantic salmon isolates represent a distinct cluster and are more closely related to the rainbow trout isolates in cluster 1 compared to the isolates represented in cluster 3. The evolutionary relationship of these *ompF* alleles is different to the isolate phylogeny (Fig. 1b) in which these Atlantic salmon isolates are less closely related to the rainbow trout isolates represented in cluster 1. The non-congruence of the *ompF* and housekeeping gene trees suggests that HGT has likely influenced the evolution of *ompF*. Cluster 3 comprises the remaining five isolates (RD162, RD354, RD64, RD28 and RD150) which are represented by five distinct lineages (*ompF3.1* to *ompF3.5*; Fig. 3b). These isolates represent diverse serotypes (O2, O5, O6 and O7) and OMP-types (1a, 2a and 2c) and have OmpF proteins (OmpF.3 to OmpF.7) of molecular masses 39,969, 40,067, 40,099, 40,128 and 40,334 Da respectively.

Amino acid sequence variation of OmpF was also greater than that of OmpA. Seven major OmpF variants (OmpF.1 to OmpF.7) were identified and amino acid variation, together with the β-barrel transmembrane domains and loop-regions, are shown in Supplementary Fig. S2. OmpF has eight surface-exposed loops (compared to the four of OmpA) and almost all of the amino acid variation occurs within these loops (Supplementary Fig. S2). Amino acid variation occurred in all loops with the exception of loop 1. Loop 3 was also relatively conserved: a single amino acid substitution at site A141G in strains RD354 and RD162. This finding is in agreement with those in other members of the Enterobacteriaceae^85^. This loop is located inside the β-barrel and plays an important role in constricting the channel, resulting in selectivity of the pore^18^. The amino acid variation in loops two, four, five, six, seven and eight was restricted mainly to six hypervariable domains, HV1 to HV6, that are located at or towards the tips of the loops (Fig. 3b) suggesting an important role in host–pathogen interactions. Hypervariable domain 1 (HV1) contained three amino acid substitutions (S91G, T92D, E93K). The most variable loops were loops four (HV2), five (HV3) and seven (HV5). In HV2 (loop 4), several amino acid replacements (D183N/K, K184D, V185T, D187V/I) were evident in strains RD28, RD64, RD150, RD162 and RD354. In HV3 (loop 5), three amino acid substitutions (V218T, A219T and S223F) and 13 amino acid insertions were present at positions 228 to 240 in the non-motile biotype 2 rainbow trout isolates RD6, RD10, RD520 and RD524 (228 N, 229R, 230 T, 231 V, 232A, 233Q, 234 K, 235D, 236S, 237 T, 238 T, 239A, 240D). Since isolate RD524 represents a newly emergent serotype O8 rainbow trout isolate, this finding suggests that the emergence of the O8 serotype in rainbow trout occurred after the insertion of these 13 amino acids in OmpF. In HV4, three amino acid substitutions (K276E, D278S and D279N) and one insertion (F227) were observed. In HV5 (loop 7), five amino acid substitutions (F325N, G326S, A327I, T328Y and A330N) and three deletions (P331, I332 and G333) occurred exclusively in isolate RD162. The variation in loops two (nt: 16.7%; aa: 25.0%), four (nt: 27.08%; aa: 25.0%), five (nt: 6.4%; aa: 14.3%), six (nt: 6.7%; aa: 0.0%), seven (nt: 18.3%; aa: 25.0%) and eight (nt: 13.7%; aa: 23.5%) were much greater than that of the membrane spanning regions (nt: 1.3%; aa: 2.0%), suggesting that natural selection is driving diversification of the loops in OmpF.

The observed diversity in *ompF* (and OmpF) between isolates is striking and suggests that amino acid variation within the surface-exposed loops of OmpF is advantageous to *Y. ruckeri*; these domains likely play an important role in some aspect of this pathogen’s biology, such as in adherence or evasion of the host immune response. Indeed, the surface-exposed loops of OMPs are involved in many host and environmental interactions^37–40^. Such diversification of the loops of OmpF has also been described in other Gram-negative bacteria including *Neisseria meningitidis*^72,73^, *Yersinia* sp.^48^, *E. coli*^49^, *P. multocida*^86^, *Haemophilus influenzae*^87–89^ and *S. Typhi*^50^ and is thought to be important in providing these pathogens with antigenic variation against the the host immune response. These surface-exposed loops are thought to interact with the host immune system and, by undergoing antigenic variation, provide the bacterium with an important defence mechanism^69–73^. Additionally, OmpF has been reported to be a protective antigen against some bacterial infections^51,64,65^. The loops of OmpF contain putative antigenic epitopes^51,52^ and indeed, a recombinant OmpF subunit vaccine provides protective immunity against *Y. ruckeri* infection in catfish^53^. The identification of a 13-amino acid insertion at the tip of loop 5 in biotype 2, serotype O1 isolates of *Y. ruckeri* could be important if this segment of the loop represents a highly immunogenic and protective epitope. Since available vaccines are based on biotype 1 or 2 strains, selection of the correct vaccine could be more important than previously thought as a means of providing protection against ERM.

## Conclusions

In conclusion, isolates of *Y. ruckeri* recovered from infected Atlantic salmon and rainbow trout represent distinct phylogenetic lineages based on the concatenated sequences of 19 housekeeping genes. However, the apparent phylogenies of certain isolates has been influenced by horizontal gene transfer and recombinational exchange. We have used splits decomposition analysis to demonstrate a net-like phylogeny based on the housekeeping genes which is characteristic of recombination. In particular, comparative analysis of the distribution of individual housekeeping gene alleles across the isolates demonstrated evidence of genomic mosaicism and recombinational exchange involving Atlantic salmon and two rainbow trout isolates. Our previous hypothesis that the novel serotype O8 has likely arisen in Atlantic salmon and subsequently been acquired by rainbow trout as a consequence of HGT is supported, as this serotype is present in divergent Atlantic salmon and rainbow trout lineages. Subtle variation was observed in OmpA, with diversity present exclusively in the surface-exposed loop 3 of this protein. Notably, isolates of divergent and pathogenic Atlantic salmon and rainbow trout isolates of serotypes O1 and O8 had identical OmpA sequences, suggesting partial or complete horizontal transfer of the *ompA* gene and a potential role for OmpA (possibly involving loop 3) in the pathogensis of *Y. ruckeri*. The OmpF protein displayed greater variation than that of OmpA. Again, amino acid variation was confined almost entirely to the loop regions and there was evidence of HGT of *ompF* between pathogenic Atlantic salmon and rainbow trout isolates suggesting a potential role in virulence. The variation observed within the surface-exposed loop regions of OmpA and OmpF, together with evidence for the HGT and selection of *ompA* and *ompF* alleles between pathogenic Atlantic salmon and rainbow trout isolates, suggests an important role for these proteins in the biology of *Y. ruckeri* that warrants further investigation. In particular, identification of the precise roles of these proteins in host–pathogen interactions could lead to the development of improved disease prevention strategies.

## Supplementary Information

Supplementary Figure S1.

Supplementary Figure S2.

Supplementary Figure S3.

Supplementary Figure S4.

Supplementary Figure S5.

Supplementary Information.
