# Supplementary figures and images for: Diversification of OmpA and OmpF of Yersinia ruckeri is independent of the underlying species phylogeny and evidence of virulence-related selection
Source: Sci Rep. 2021 Feb 10;11:3493. doi: 10.1038/s41598-021-82925-7 (PMC7876001; doi:10.1038/s41598-021-82925-7)

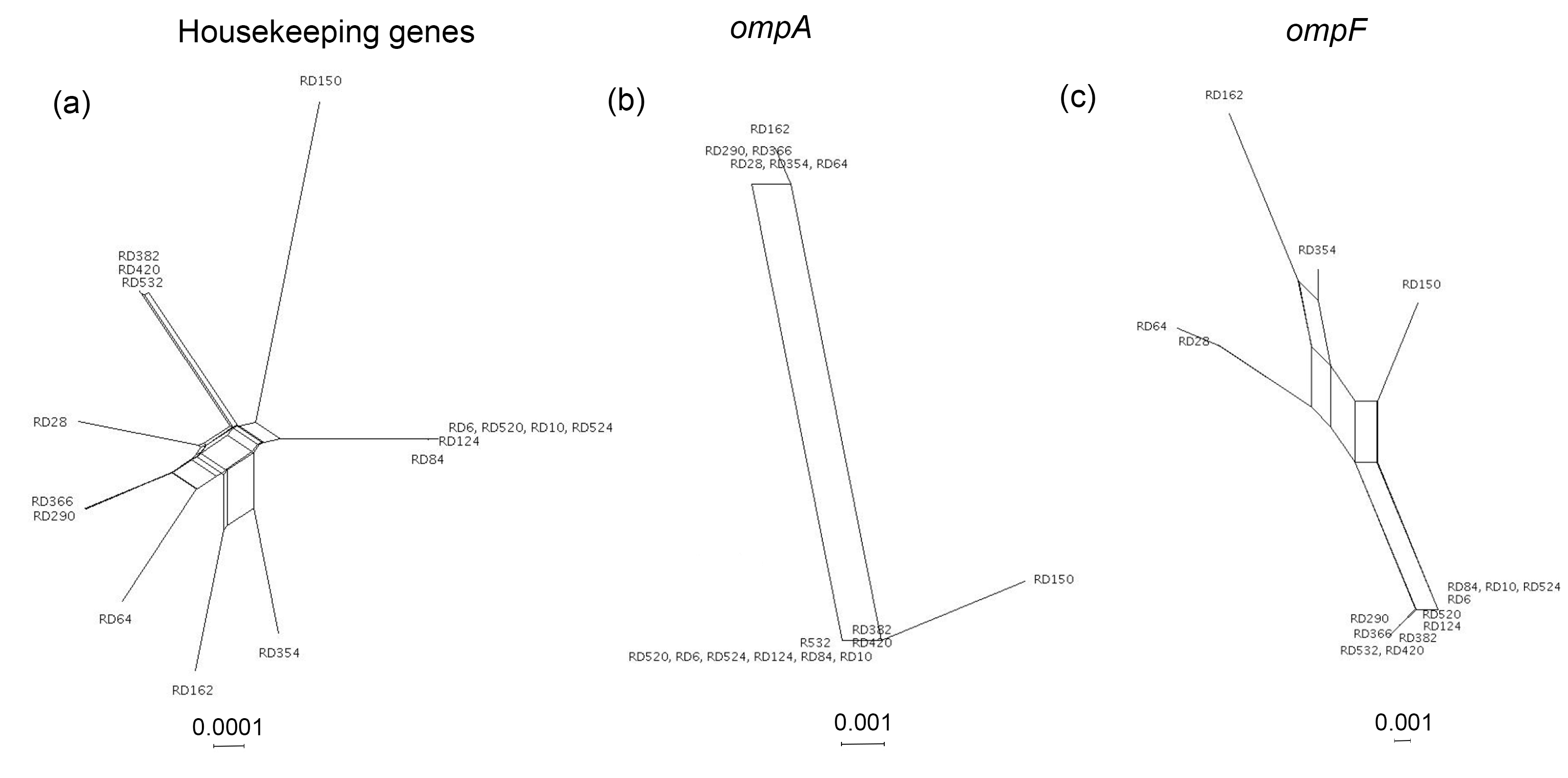

Supplement: Supplementary file 1 — Supplementary Figure S1. [file 41598_2021_82925_MOESM1_ESM.tif]

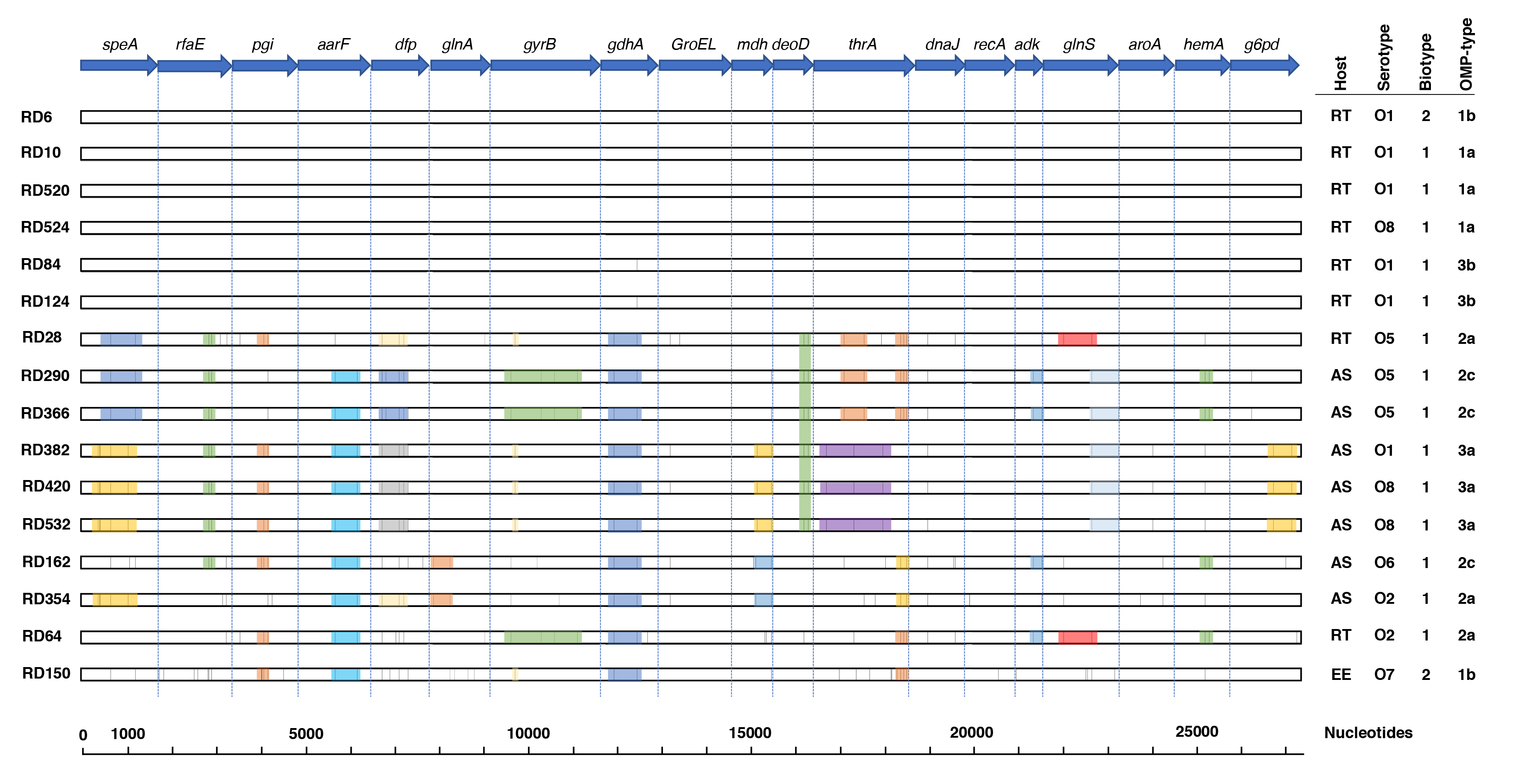

Supplement: Supplementary file 2 — Supplementary Figure S2. [file 41598_2021_82925_MOESM2_ESM.tif]

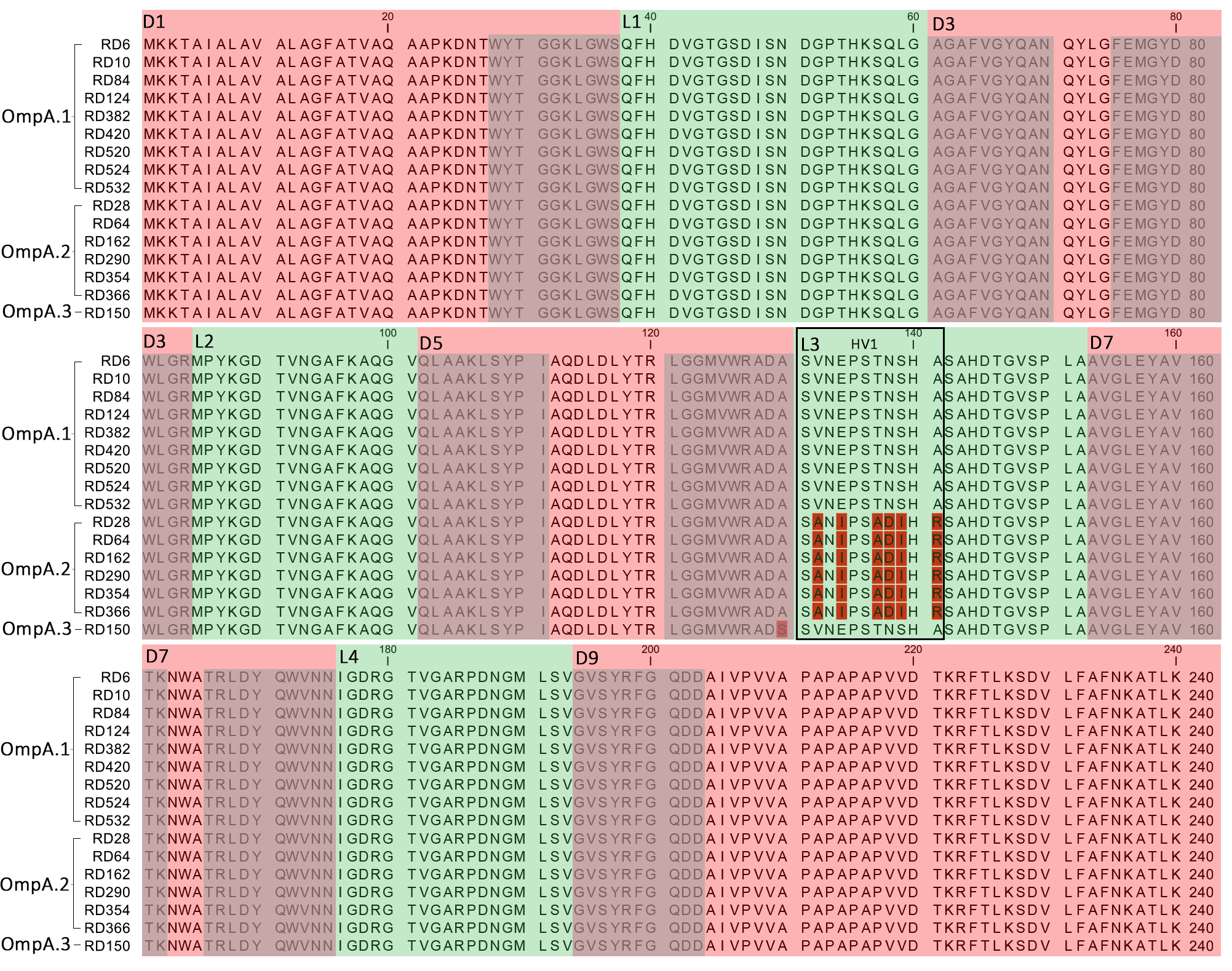

Supplement: Supplementary file 3 — Supplementary Figure S3. [file 41598_2021_82925_MOESM3_ESM.tif]

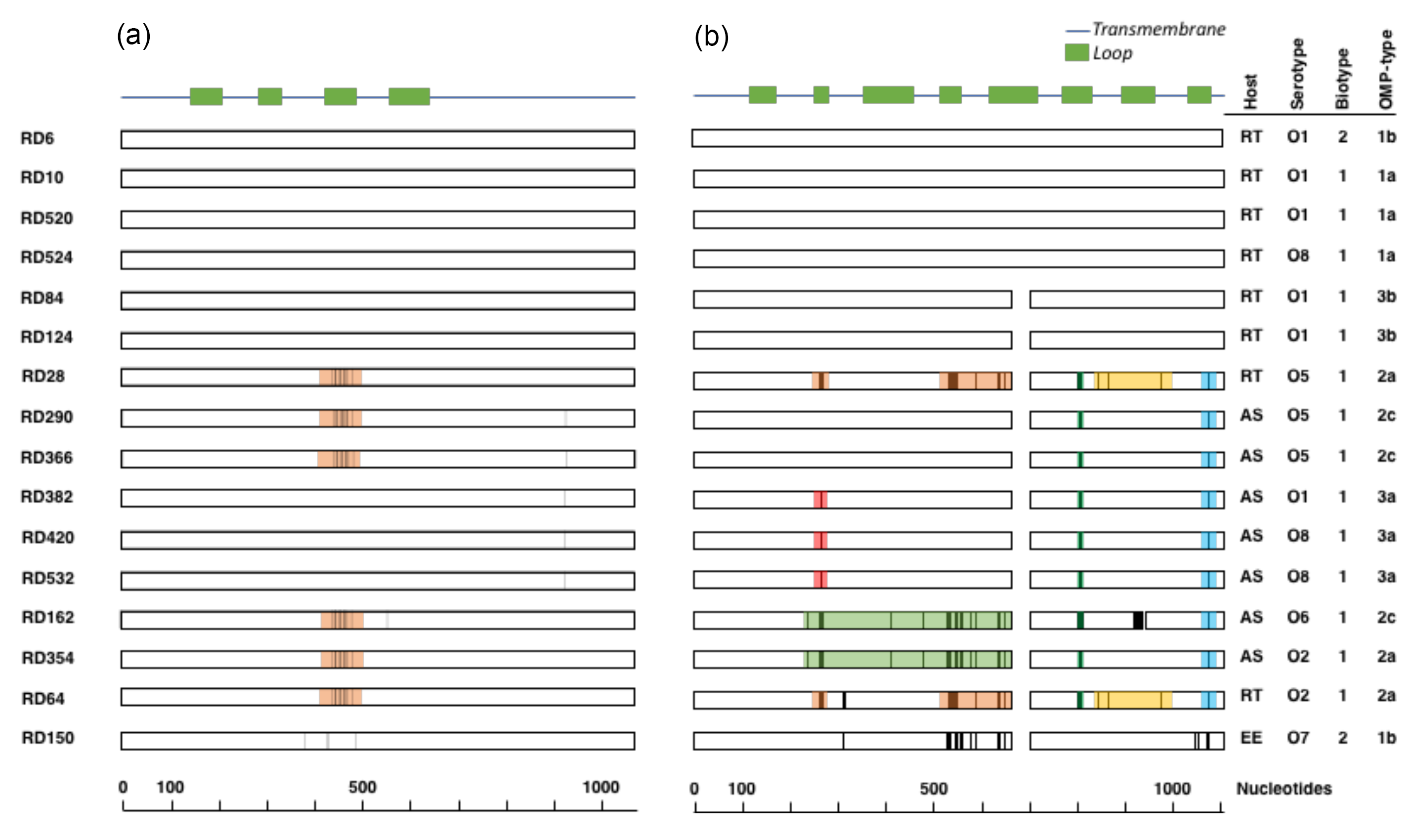

Supplement: Supplementary file 4 — Supplementary Figure S4. [file 41598_2021_82925_MOESM4_ESM.tif]

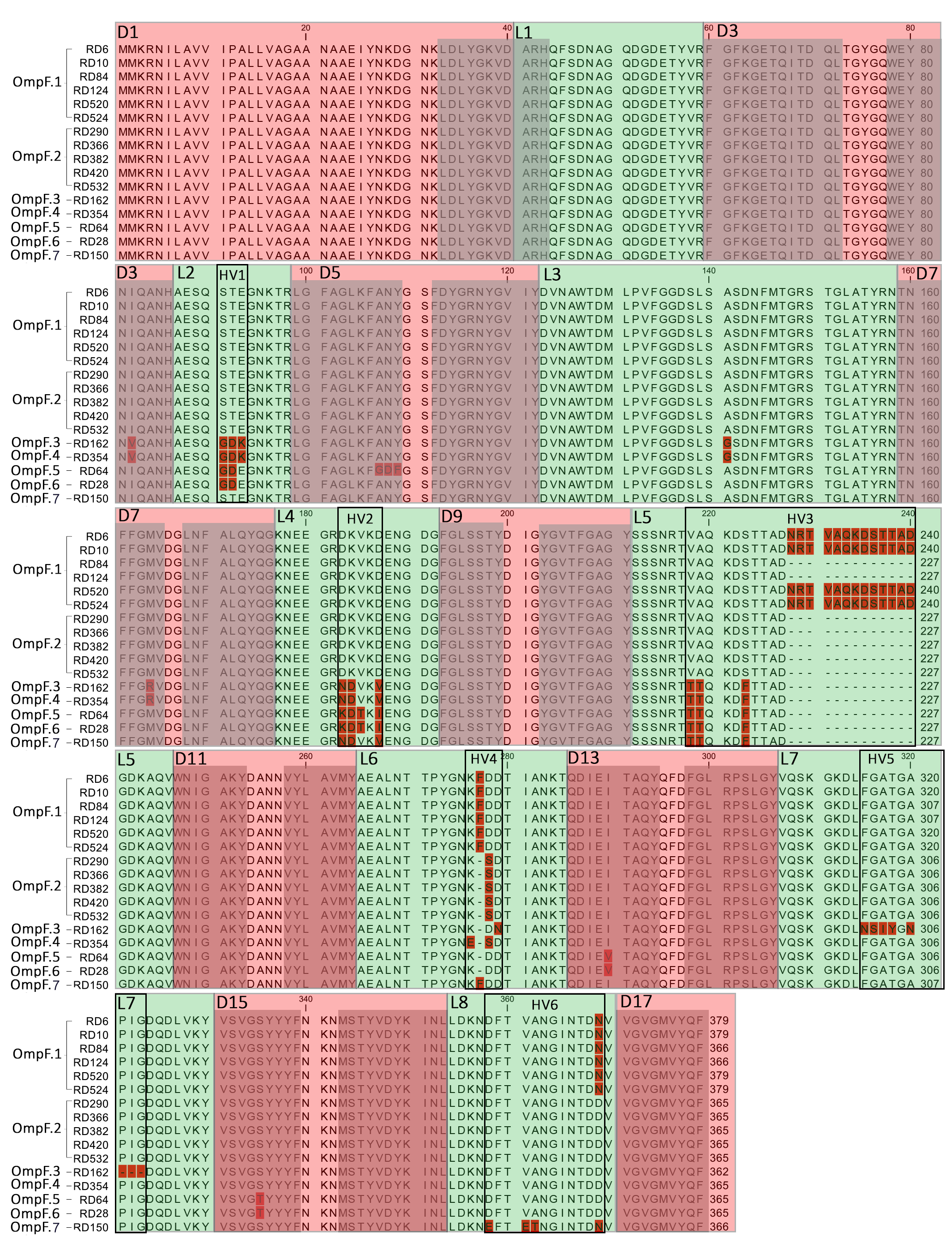

Supplement: Supplementary file 5 — Supplementary Figure S5. [file 41598_2021_82925_MOESM5_ESM.tif]
