# Supplementary material for: Diversification of OmpA and OmpF of Yersinia ruckeri is independent of the underlying species phylogeny and evidence of virulence-related selection
Source: Sci Rep. 2021 Feb 10;11:3493. doi: 10.1038/s41598-021-82925-7 (PMC7876001; doi:10.1038/s41598-021-82925-7)

**Title**

**Diversification of OmpA and OmpF of *Yersinia ruckeri* is independent of the underlying species phylogeny and evidence of virulence-related selection**

**Authors**

Michael J. Ormsby***** and Robert L. Davies

**Affiliations**

*^1^Institute of Infection, Immunity and Inflammation, College of Medical, Veterinary and Life Sciences, Sir Graeme Davies Building, University of Glasgow, Glasgow G12 8TA, United Kingdom*

**Lead contact email address:*

[*Michael.ormsby@glasgow.ac.uk*](mailto:Michael.ormsby@glasgow.ac.uk)

**Lead contact address:*

Dr. Michael J Ormsby
Institute of Infection, Immunity and Inflammation
College of Medical, Veterinary and Life Sciences
Sir Graeme Davies Building
University of Glasgow
120 University Place
Glasgow G12 8TA

**Supplementary Fig. S1 Splits decomposition analysis of housekeeping genes, *ompA* and *ompF* of *Y.* *ruckeri*.** Splits decomposition trees were constructed for the concatenated housekeeping genes (a), *ompA* (b) and *ompF* (c) with SplitsTree 4.0 (http://www.splitstree.org) to test for recombination ^59^ which is indicated by the net-like phylogenies.

**Supplementary Fig. S2 Schematic representation of nucleotide variation in the concatenated housekeeping gene sequences of 16 *Y.* *ruckeri* isolates**. Nucleotide variation at single sites with respect to isolate RD6 (top-most isolate) is represented by vertical lines within housekeeping genes from each isolate. The different colors highlight sequence identity between isolates. Gene designations and positions within the concatenated sequence are indicated at the top of the diagram. Numbers along the bottom indicate the nucleotide position within each concatenated sequence. Strain designations are provided in the left-hand column.. RT – Rainbow trout; AS – Atlantic salmon; EE – European eel.

**Supplementary Fig. S3 Distribution of variable inferred amino acid sites in the N-terminal transmembrane domains of OmpA.** The numbers above the sequences represent amino acid positions. Loops are indicated by L1-4 and are highlighted in green. Other domains are indicated by D1-D9 and are highlighted in red. Transmembrane β-sheets within each domain are highlighted as shaded regions. HV represents the hypervariable domain within surface-exposed loops 1 to 4. Domains were predicted using Pred TMBB based on Hidden Markov Models (HMM). Distinct variants are represented by OmpA.1 to OmpF.3 and reflect those in Fig. 2.

**Supplementary Fig. S4 Schematic representation of nucleotide variation in the *ompA* and *ompF* genes of 16 *Y. ruckeri* isolates**. Nucleotide variation at single sites with respect to isolate RD6 (top-most isolate) is represented by vertical lines within ompA (a) and ompF (b) alleles from each isolate. The different colors highlight sequence identity between isolates. The locations of transmembrane regions and surface-exposed loops are represented at the top of the diagram. Numbers along the bottom indicate the nucleotide positions within each gene. Strain designations are provided in the left-hand column. RT – Rainbow trout; AS – Atlantic salmon; EE – European eel.

**Supplementary Fig. S5 Distribution of variable inferred amino acid sites in the N-terminal transmembrane domains of OmpF.** The numbers above the sequences represent amino acid positions. Loops are indicated by L1-8 and are highlighted in green. Other domains are indicated by D1-D17 and are highlighted in red. Transmembrane β-sheets within each domain are highlighted as shaded regions. HV represents the hypervariable domain within surface-exposed loops 1 to 8. Distinct variants are represented by OmpF.1 to OmpF.7 and reflect those in Fig. 3. Domains were predicted using Pred TMBB based on Hidden Markov Models (HMM).

**Supplementary Table S1 Genome coverage of the sequenced samples**

| **Sample Name** | **Sequence reads (millions)** | **Coverage** |
| --- | --- | --- |
| **RD6** | 0.6 | 159.8 |
| **RD10** | 0.6 | 159.8 |
| **RD124** | 0.6 | 159.8 |
| **RD150** | 1.1 | 160.3 |
| **RD162** | 1 | 160.2 |
| **RD28** | 0.7 | 159.9 |
| **RD290** | 1.1 | 160.3 |
| **RD354** | 1 | 160.2 |
| **RD366** | 1 | 160.2 |
| **RD382** | 1 | 160.2 |
| **RD420** | 1.1 | 160.3 |
| **RD520** | 0.9 | 160.1 |
| **RD524** | 0.9 | 160.1 |
| **RD532** | 0.9 | 160.1 |
| **RD64** | 0.9 | 160.1 |
| **RD84** | 0.7 | 159.9 |

**Supplementary Fig. S1**


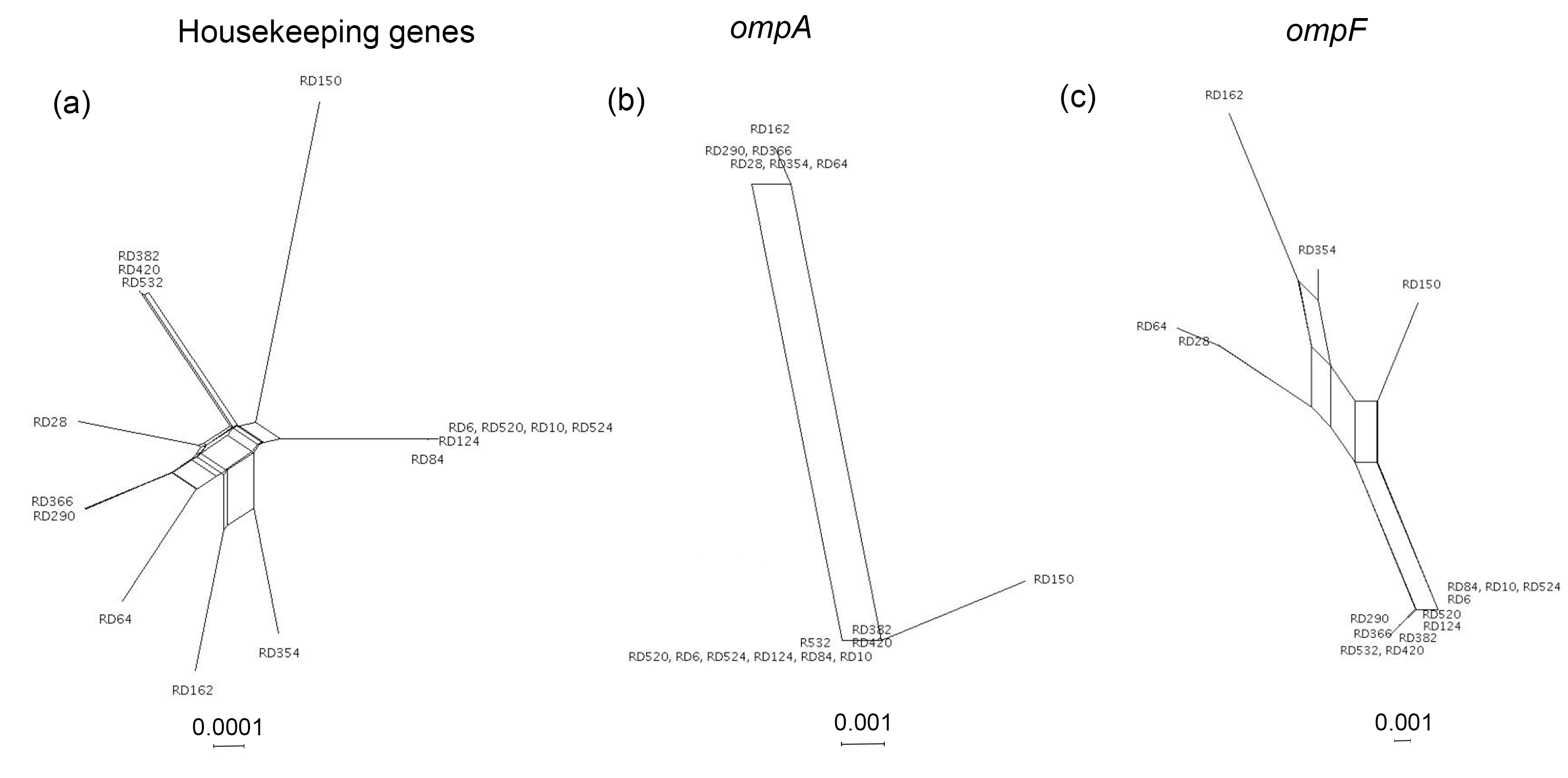


**Supplementary Fig. S2**


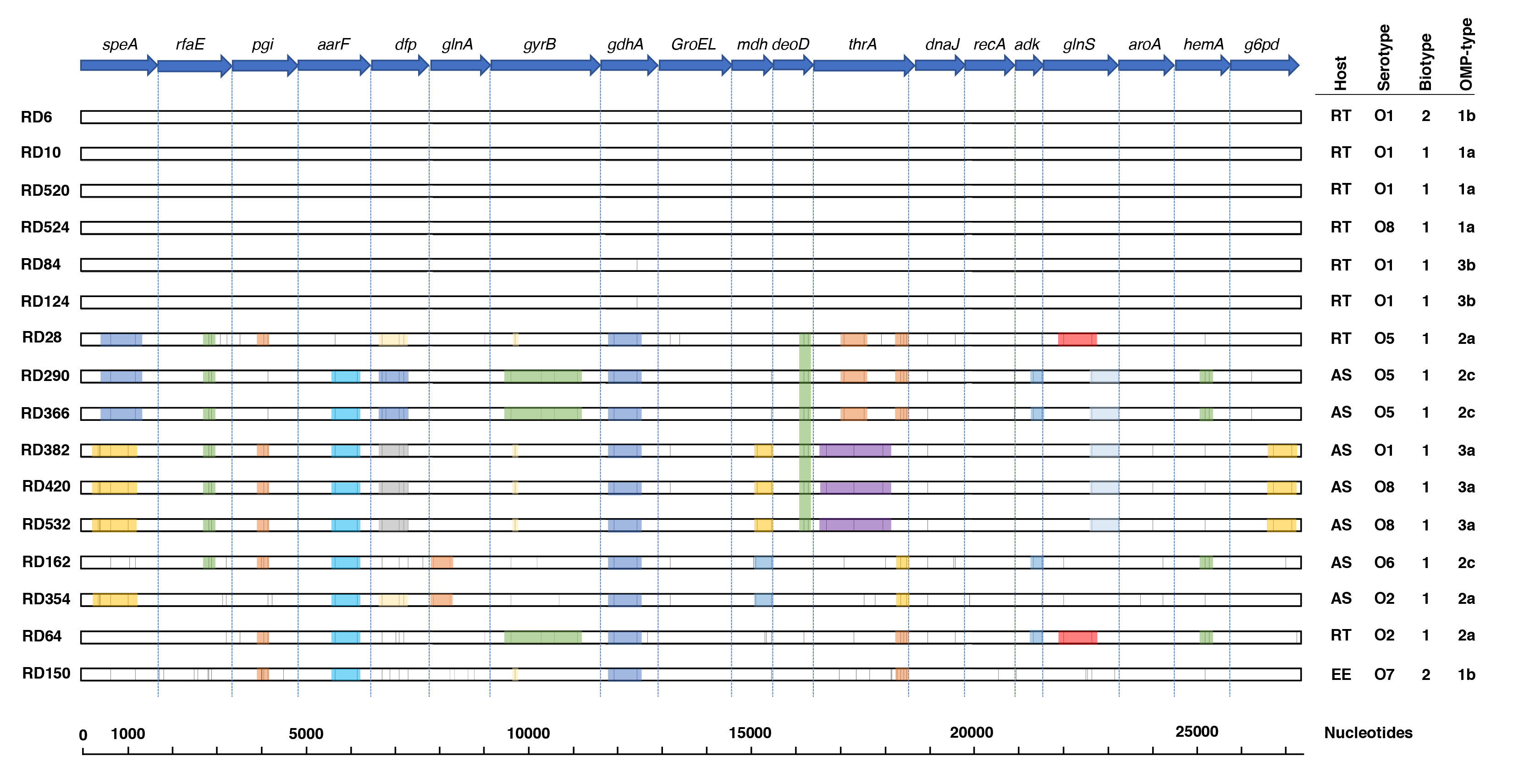


**Supplementary Fig. S3**


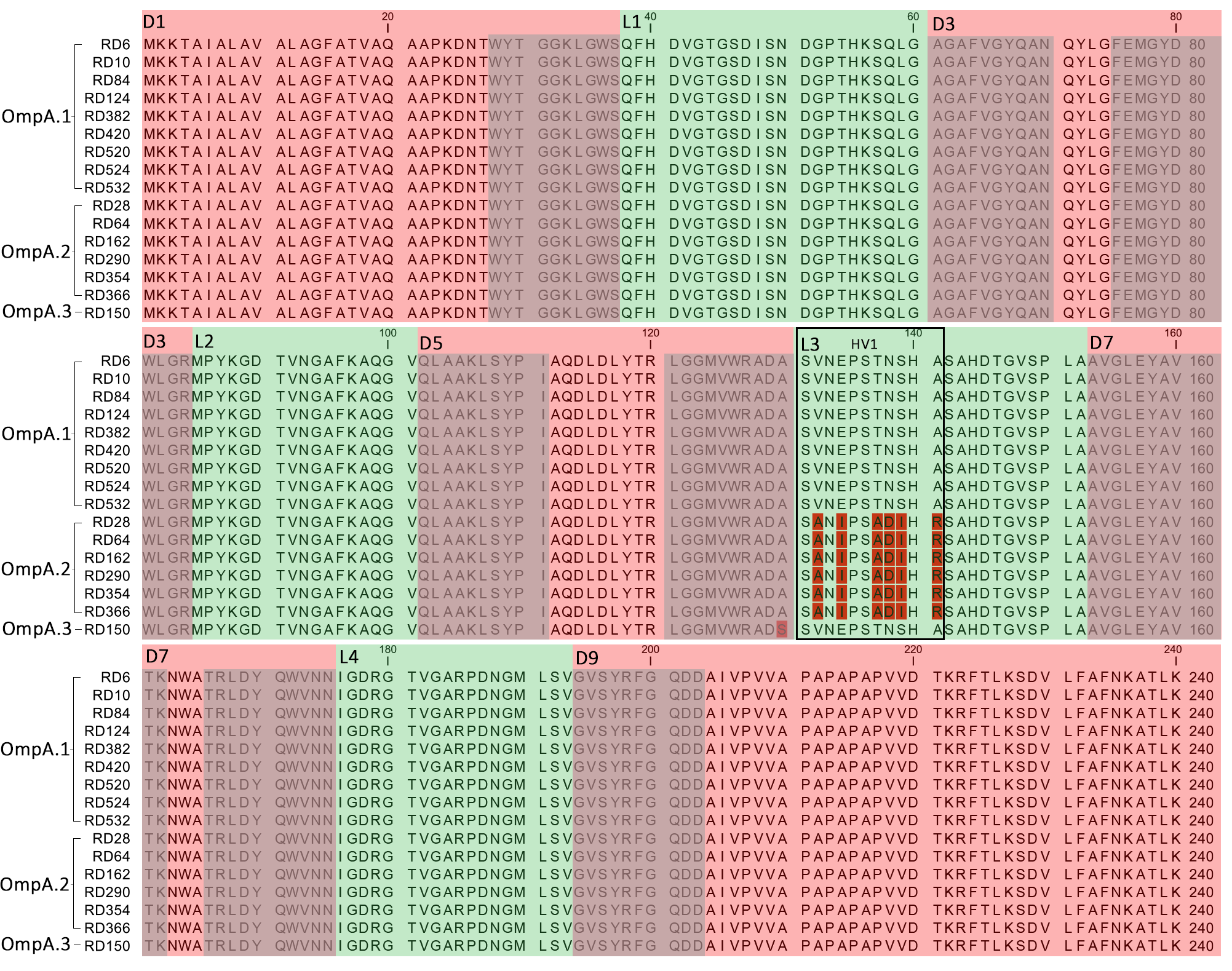


**Supplementary Fig. S4**


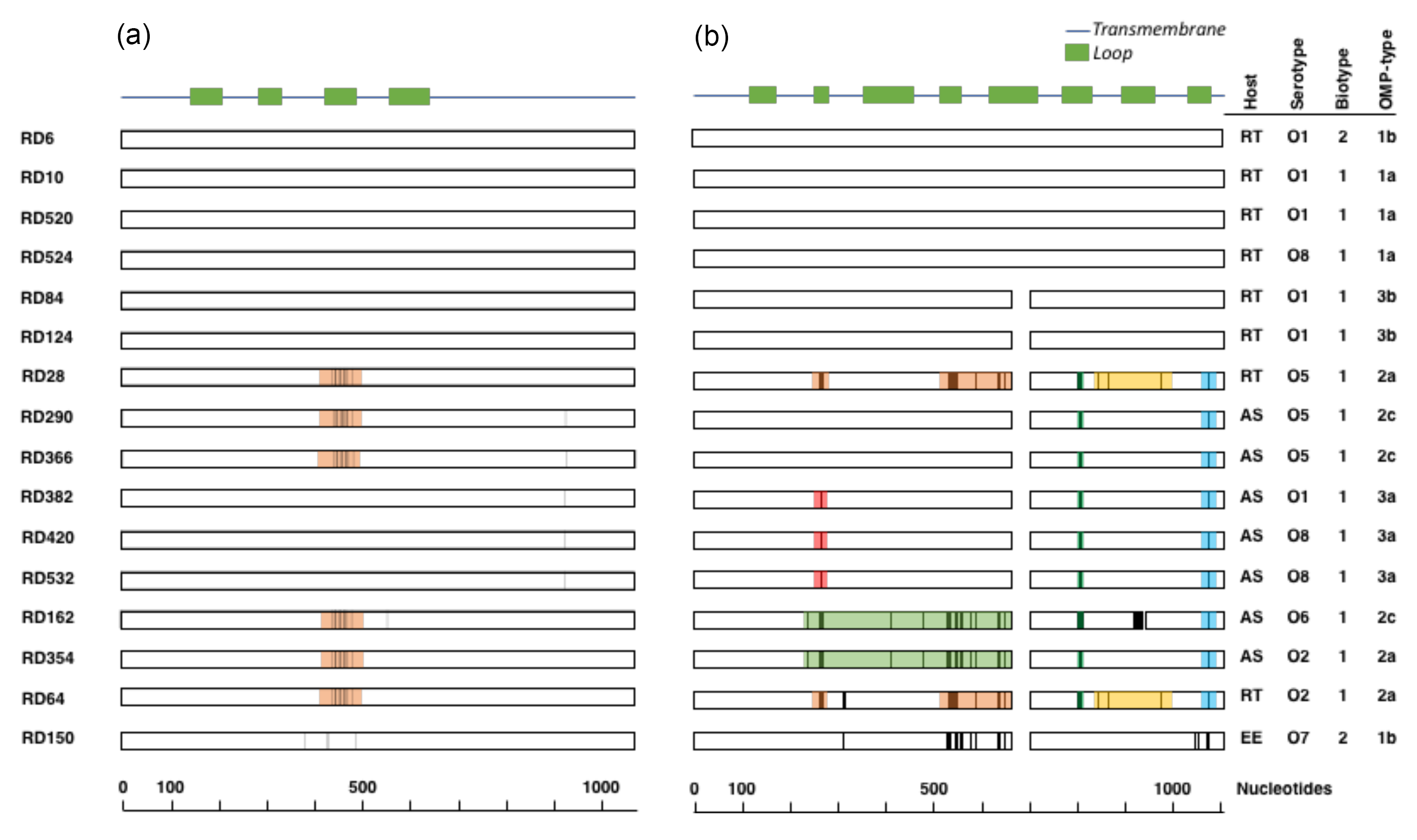


**Supplementary Fig. S5**


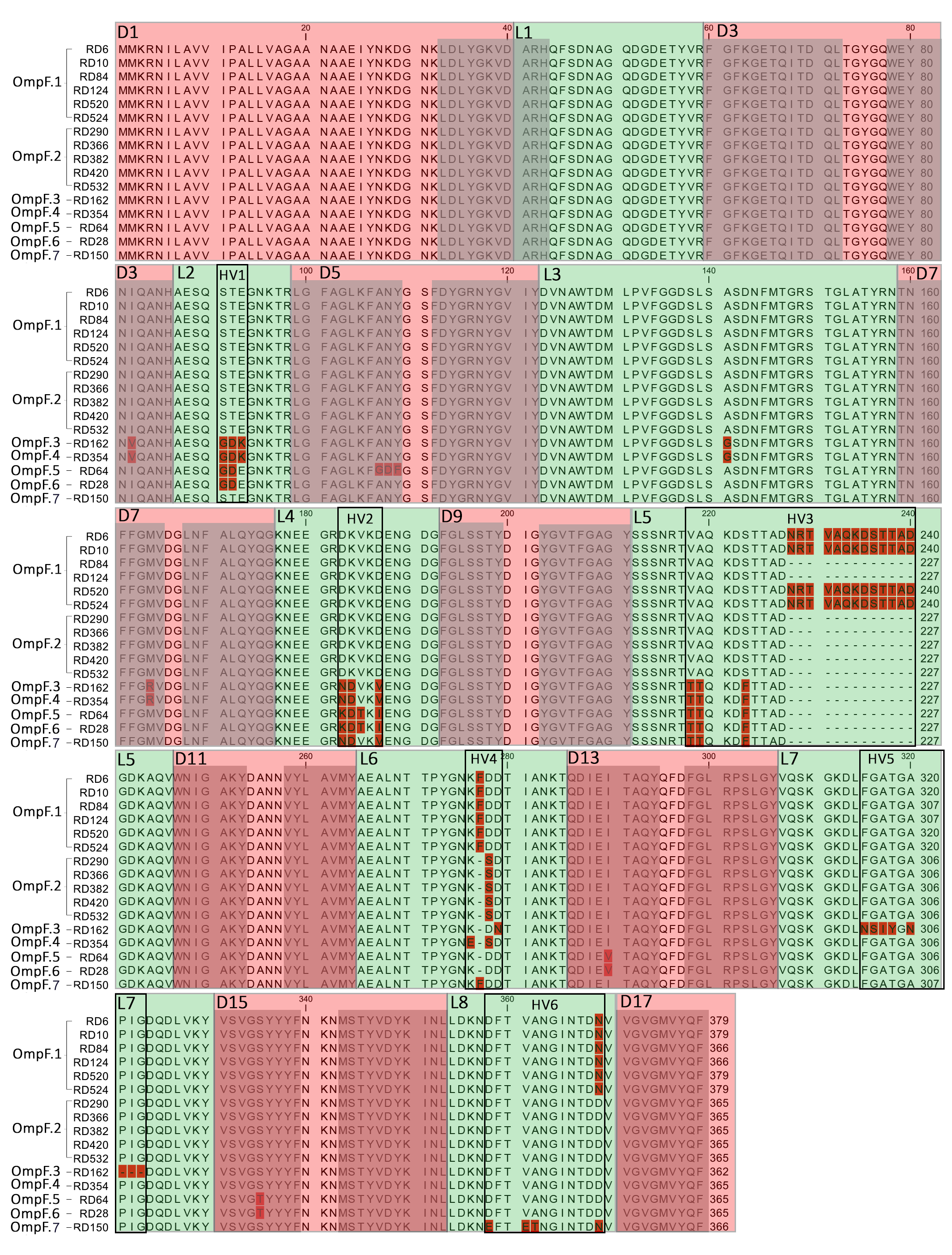

Supplement: Supplementary file 6 — Supplementary Information. [file 41598_2021_82925_MOESM6_ESM.docx]
